# The Prevalence of Unused Medications in Homes

**DOI:** 10.3390/pharmacy7020061

**Published:** 2019-06-13

**Authors:** Mutaseim Makki, Mohamed Azmi Hassali, Ahmed Awaisu, Furqan Hashmi

**Affiliations:** 1 School of Pharmaceutical Sciences, University Sains Malaysia, Penang 11700, Malaysia; azmihassali@usm.my; 2 College of Pharmacy, Qatar University, Doha 2713, Qatar; aawaisu@qu.edu.qa; 3 College of Pharmacy, University of the Punjab, Lahore 54590, Pakistan; furqan.pharmacy@pu.edu.pk

**Keywords:** unused medications, home, prevalence, non-adherence, wastage, disposal

## Abstract

The prevalence of unused medications in homes has dramatically increased in recent decades, which has resulted in medication wastage. The aim of this study is to review the prevalence of unused medications in homes and to determine the reasons behind this disuse, so as to help reduce such wastage. The review also sheds light on current methods of disposal of unwanted medications. Here, using a narrative review, we provide an overview of the issues of unused medications, medication wastage, and methods of disposal. We conducted an extensive literature search focusing on subject-related keywords, as given in the methods section below. A search was undertaken through indexing services available in the library of the authors’ institution. Full-text papers concerned with the prevalence of unused medications in homes, written in English language between 1992 and 2018, were retrieved and reviewed. Twenty-five related studies performed in different world regions were reviewed and included. The public, healthcare providers, and governments are all accused of promoting medication wastage in different ways, and thus, they need to be targeted to solve the problem. It was also noticed that the prevalence of unused medications is high in many countries. Non-steroidal anti-inflammatory drugs are among the most frequently wasted medications, and most of the public just dispose of their expired medications in the trash or toilet. Non-adherence, death, and medication change are among the main causes of medication accumulation and consequent wastage. A lack of policies to return unwanted medications in some countries, as well as public unawareness, carelessness, or illiteracy, are reasons for improper disposal of unused medications that may lead to adverse economic and environmental impacts. Various mitigation strategies (e.g., smart medicine cabinet) have emerged to reduce medication wastage. Joint work among the public, healthcare providers, and various governmental and private organizations is needed to adequately address the issue of medication wastage.

## 1. Introduction

The accumulation of unused medications leads to medication wastage and causes consequent losses of economic resources. Healthcare systems strive to offer patients the best quality of service, for example, through maximum utilization of the available resources, while simultaneously minimizing any possible monetary losses [1]. One definition of waste is, “Any substance or object the holder discards, intends to discard, or is required to discard” [2]. Unused medications include expired, spilt, and contaminated pharmaceutical products, drugs, vaccines, and sera that are no longer required and need to be disposed of appropriately [2]. Although the issue of unused medications is well-defined and known all over the world, there are still obstacles to the reduction of medication wastage. Lots of mitigation approaches that involve the reduction of wastage by pharmacists, prescribers, and the public have been set out in the developed world. To ensure the appropriate use of finite resources, it is necessary to reduce any wastage, including that of medication [3]. In different regions of the world, medication wastage has become a burden on the fiscally restrained healthcare systems. In many cases, economic loss caused by medication wastage could have been avoided if appropriate measures had been taken, and the resultant economic savings would have benefited other healthcare schemes [1]. A similar report stated that this waste represents a lost opportunity to improve the health outcomes of the patients involved, as well as a waste of health budget resources that could have been used to fund other much-needed areas of healthcare [4]. Medication wastage has considerable economic and environmental impacts, as it causes pollution. The total value of medicines returned to three tertiary hospitals exponentially increased from the baseline value of RM 27,899, to RM 53,769, and then to RM 190,616 after the Ministry of Health in Malaysia launched the “Return Your Medicines Program” in 2010 [5]. In 2006, Ekedahl found that in Sweden, two reasons appear to account for 71% of all medicine returns to pharmacies, namely patient deaths (45%) and therapy changes (26%) [4]. In that study, Ekedahl’s comparison showed that the expenses lost in programs to minimize medication wastage is higher than the expenses spent to procure the same medications. This loss would have been justified if patients had received more healthcare benefits [4]. The World Health Organization (WHO) recommends that unwanted medications should never be used and should always be considered as pharmaceutical waste [6]. The United States of America is considered to be the world’s top medical waste-producing nation, with an annual production of over 3.5 million tons of medical waste and an average disposal cost of $790 per ton [7]. The 1988 Medical Waste Tracking Act (MWTA) of the United States is a legislation governing American medical waste that provides high quality medical waste regulation by defining and assorting medical waste subject to program regulations [8]. The MWTA aims to

- “Establish a cradle-to-grave tracking system utilizing a generator-initiated tracking form”;
- “Maintain management standards for segregation, packaging, labelling, and storage of the medical waste”;
- “Establish recordkeeping requirements and penalties that could be imposed for mismanagement” [8].

Bakiu et al. interviewed Albanian citizens who pointed out that there is no proper management of the medical waste from the relative producer. Thus, the first actions needed are the training of inspectors and the establishment of a subvention system in the private and state healthcare systems [9]. Many regions of the world, including countries in the Middle East, such as the Sultanate of Oman, lack national programs for the return of unwanted medications [10].

The combination of unnecessary prescriptions, together with low medication adherence, silently contributes to the waste of finite resources, which further accentuates the already rising healthcare costs [11]. In 2014, the “WHO” Medication Situation Report stated that medication adherence is about 50% worldwide, and it is lower in developing and transitional countries [12]. In the World Medicines Situation 2011, Holloway stated that the irrational use of medicines is an extremely serious global problem that is wasteful and harmful. Holloway further added, “in primary care, less than 40% of patients in the public sector and 30% of patients in the private sector are treated in accordance with standard treatment guidelines in developing and transitional countries” [13]. An observational study in Qassim province, Saudi Arabia, revealed that the percentages of unused analgesics, antibiotics, and vitamins are 50.5%, 32.5%, and 6%, respectively [14]. Medical waste is increasingly being formed by the introduction of supposedly safer single-use medical devices, which are on trial to replace the multi-use ones. This leads to unsafe disposal due to the accumulation of a high volume of these devices [15]. A group of researchers directly associated the increased number of drugs in homes to household size [15]. 

It is clear that medication wastage is associated with some drawbacks from economic and environmental points of view. Economically, the drug supply chain will not be cost-effective, as after the complex processes conducted to professionally prepare, store, and deliver these medications to patients, the end-products remain unused. From the environmental viewpoint, wasted medications adversely affect humans, animals, and plants if they are not safely disposed of [16]. 

In the State of Qatar, Kheir et al. stated, “Many countries limit the list of Over-the-Counter (OTC) medicines to those with a proven safety profile, while extending the prescription-only list to include all medications that could be potentially harmful” [17]. Other countries limit the prescription medication list, while making a long list of OTC products available. This is exemplified by the community’s underestimation of the pharmacist’s role in educating patients and by insufficient pharmacist–patient interaction, as shown by another study in Qatar [18].

There are different mitigation strategies that may reduce medication wastage, such as curbing the waste of anticancer medications by batching patients (i.e., scheduling a group of patients to a medication vial at the same time) to facilitate vial sharing and rechecking the expiry dates to ensure these medications are consumed before it is too late [19]. Expiration of pharmaceuticals was shown to be the main cause of medication wastage and subsequent disposal in a study done in Florida in the United States [19]. Management of scheduled removal of nearly-expired medications from pharmacies and long-term care units would help to reduce medication wastage [19].

Direct advertisement of medications is considered unethical in many regions of the globe, but in 1997, the United States Food and Drug Administration (US FDA) legalized these advertisements to some extent, which lead to the expansion of medication wastage. In an attempt to diminish medication wastage, the FDA has put forward certain suggestions [19], including:

- Formulating and executing continuing education programs for healthcare providers on topics related to the reduction of medication, e.g., educating them about the effects of medication wastage, improper disposal, and the unfavorable impact on the environment.
- Restricting distribution of free medication samples and substituting them with vouchers.
- Making the patients’ prescriptions accessible by authorized professional personnel.
- Permitting patients to partially fill up a prescription, so as to not overstock medications in homes.
- Curtailing automatically refilled prescriptions to some extent.
- Providing patients with a trial quantity of medications until adherence is assured.
- Removing the hassle that contributes to medication wastage by renewing and updating regulations at all levels.
- Notifying prescribers of the toxic effects of wasted medications through common electronic platforms.

One of the mitigation strategies is to assign a small number of medicines to patients and increase the quantity gradually [20]. In the same research paper, it was suggested that pharmacists could supply a trial quantity of medicine until they confirm its suitability for the patients, thus circumventing medication wastage [20]. Other mitigation approaches that can contribute to medication saving are split-filling the supply, increasing the frequency of medication batch preparations, or simultaneously dispensing the same medications prescribed to a group of patients on a specific date from a hospital pharmacy. In light of the aforementioned literature, we aim to review the prevalence of unused medications in homes and to determine the reasons behind this disuse so as to help reduce such wastage. The review also sheds light on current methods of disposal of unwanted medications.

## 2. Materials and Methods

**Figure 1:**
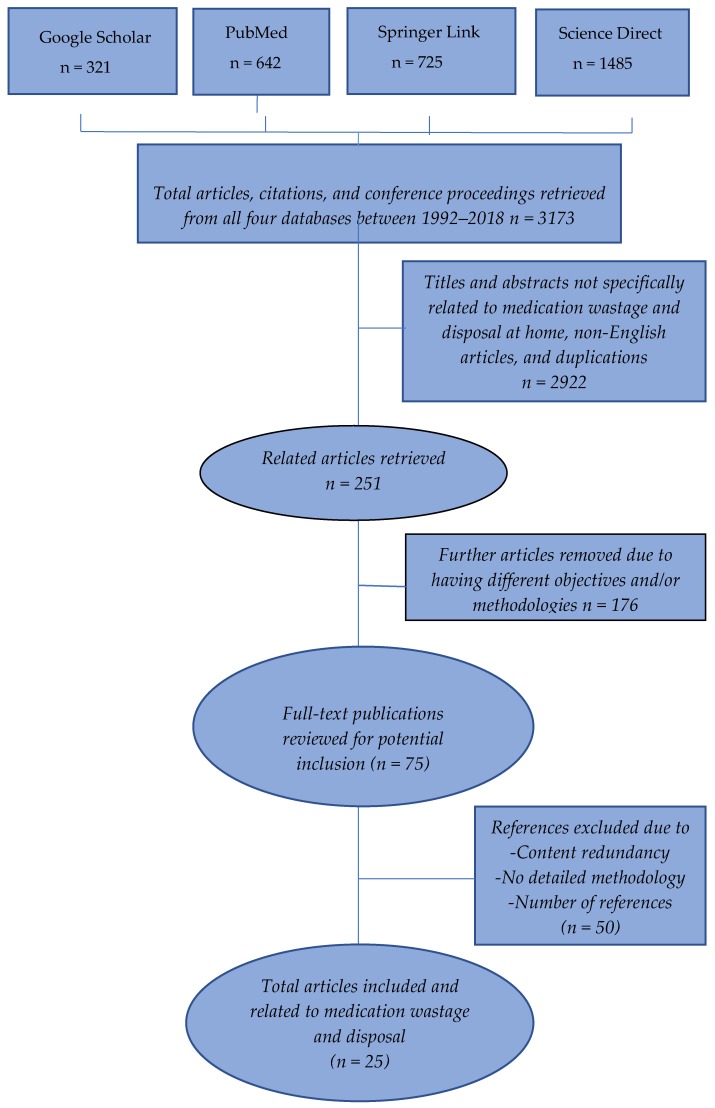
Quorum flow chart for article selection.

This narrative review uses a qualitative approach to conduct a critical analysis of the literature published in books and electronic or paper-based journal articles. Two authors conducted a comprehensive query of the electronic databases PubMed, Google Scholar, Springer Link, and Science Direct to search literature related to the prevalence of unused medications in homes. The search was done between July 2018 and May 2019 using the keywords “unused”, “unwanted”, “expired”’, “house”, “home”, “residential”, “medications”, “medicines”, “pharmaceuticals”, “drug”, “wastage”, “waste”, “disposal”. Firstly, articles published from 1992 to 2018 containing at least one of the keywords were chosen. The search strategy involved the use of Boolean operators, i.e., “AND”, “OR”, and “NOT”, which can either narrow or broaden the search records. Medical Subject Headings (MeSH), thesaurus browsers, and MeSH on Demand were used whenever possible. Regardless of the articles’ authors or publication journals, any full-text article published in English was chosen. Using Zotero 5.0 (Zotero is a reference and citation manager project of the Corporation for Digital Scholarship and the Roy Rosenzweig Center for History and New Media, USA), the in-text citations and bibliography were organized. At first, 3173 articles, citations, or conference proceedings were found on the four searched websites. Articles related to non-human medications, reagents, medical devices, and medication wastage in hospitals and clinics were excluded from this review. Grey articles and articles written in languages other than English were also excluded. Duplicated articles and citations were also removed to bring the total number excluded to 2922. The resultant 251 titles and abstracts were further filtered, and eventually, 176 titles with different objectives or methodologies were removed. The remaining 75 full-text articles were retrieved for further evaluation, i.e., manual validation of content redundancy, detailed methodologies, and number of references. Both primary and secondary articles handling perspectives of patients, as well as healthcare providers, on unused medications were also added. Twenty-five articles were ultimately quality-assessed and chosen. The quorum flow chart for selection in this review is shown in Figure 1 below.

## 3. Results

This review identified numerous reasons related to the idea that medication wastage needs careful professional handling. These reasons are narrated below.

### 3.1. How Medications Are Wasted

Medications are wasted because of one or more reasons attributed either to households, healthcare providers, or regulations.

#### 3.1.1. Household Causes

A major cause of medication wastage (50%) was found to be patients’ non-adherence to medication-taking. Failure to remember taking one’s own medication and irregular behavioral lifestyles came next after non-adherence as causes of medication wastage [21].

(A) Non-adherence: 

Non-adherence results when a patient does not initiate or continue medication that a provider has recommended [22]. Non-adherence occurs due to:

1. **The scarcity of patients’ awareness:** Many patients are unaware of the importance of taking their medications in a timely manner, as prescribed to them, which leads to medication wastage. A direct proportional relationship exists between awareness and medicine-taking [21].
2. **Inconvenient experiences:** Non-adherence can happen when patients find it disruptive to take certain medications, for example, young patients with type 1 diabetes mellitus who may disuse insulin injections due to fear and pain caused by the needles. Likewise, anti-retroviral medications are wasted due to stigma among HIV patients, and anti-depressants can be wasted due to rebuttal from those affected [21].
3. **Side effects:** Patients dislike side effects caused by medications, and therefore may stop using them. Patients encouraged by healthcare staff and other householders to appropriately take their medications show substantially higher adherence, especially when such encouragement is done at the start of a new regimen [21].
4. **Beliefs on medication effectiveness:** In some Mexican households, Gracia-Vásquez and Ramírez-Lara noted that many patients stop using their Non-Steroidal Anti-inflammatory Drug (NSAIDs) analgesics when they feel that they are not improving; however, NSAIDs often become less effective over time, resulting in unused medication [23]. Some patients believe choosing another traditional therapy in place of today’s medicines may give them more relief, and thus they rarely stick to prescribed treatments [21].
5. **Low self-efficacy:** Medication might become wasted when disused by individuals who think that they are capable of treating themselves in a different way without taking their medications [21]. This was evidenced in a study of HIV patients, where adherence to anti-retroviral medication was shown to be compromised due to behavioral obstacles, cognitive factors, disbelief in anti-retroviral effectiveness, substance abuse disorders, or structural obstacles, such as homelessness and lack of insurance [24].
6. **Over-confidence:** Accumulation of unused medications occurs in houses of over-confident people. These stereotyped people tend to rely on their individual actions to bypass medication taking, for example, hypertensive patients who believe that they can control hypertension without medication [21].
7. **Mental illnesses:** Depression can induce medication non-adherence [25].
8. **Influence of other household members or carers:** Contrary to what many parents may worry about, it is necessary not to underestimate children’s ability to adhere to their medications. Parents’ attitudes may complicate medication usage rather than helping children to adhere to their medications [21]. Similarly, a disabled adult should be given the opportunity to adhere to their medications instead of distrusting their adherence capability. The presence of residential mates promotes adherence as they can remind their partners to take their medications, and hence are considered helpful co-patients [21]. Married people were found to be medication-adherent [26].
9. **Lifestyles and events:** Lifestyles affect adherence to medications. Busy schedules and a fast course of life events can make some patients miss their medications doses. Contrarily, well-organized patients seldom miss or forget taking their medications and are less likely to waste medicines. Inevitable incidents are sometimes responsible for medication wastage, such as going on holiday, hospitalization, or death [21].
10. **Patient’s Age:** Geriatric and pediatric patients are known to have higher incidences of non-adherence in comparison to the rest of the population [27]. In Canada, an increasing number of elderly people are in need of greater medical care, including the use of medications, which is directly proportional to medical waste [28].
11. **Forgetfulness:** Forgetfulness has a role in missing doses of medications. For example, a study concluded that immune-compromised kidney patients need to be reminded to take their medications and also need support to alleviate their depression [29].

(B) Other Household Causes: 

Besides the reasons highlighted above, households may waste medications for the following reasons.

1. **Fear of medication shortage:** Some patients become afraid that their medications will be unavailable when they need them, and therefore, they overstock medications [30].
2. **Improper storage of medications:** Inadequate storage may spoil medications, rendering them invalid for human use, and they, therefore, become waste. The “smart medicine cabinet” is an electronic invention which is used to store medications safely, remind patients of their medication timings, and warn the user of nearly expired medications. With these features, such a device can assist in adherence, thus minimizing medication wastage [31].
3. **Medication misplacement or loss:** Missing or losing a medication can eventually lead to waste, because patients seek a replacement for the missed medication.
4. **Controversial Advertisement Influence:** Paradoxically, advertisements have both positive and negative influences on patients. They are able to promote medication adherence by reminding patients to take their medications, but advertisements can also promote unnecessary or harmful prescribing, which may lead to medication wastage. Patients may be attracted by an advertised medicine, and thus ask doctors to prescribe these medications despite their clinical inappropriateness. Patients’ dissatisfaction caused by doctor’s denial to fill a prescription may induce patients to switch doctors (thus, putting extra pressure on doctors), or they may get the medication from somewhere else [32].

#### 3.1.2. Health System Causes

1. **Repeat treatment prescribing:** Over-supply of medication can result in medication accumulation and consequent waste. This happens when prescribers unknowingly write new prescriptions to their patients without making sure or being unable to make sure whether the patient already has these medications [27,33]. In a study focused on reducing repeated prescribing of the same medications, pharmacists were shown to be of help in this regard. Their interventions help patients to avoid adverse effects, as well as drug–drug interactions [34]. Minimizing the over-supply of medications would reduce medication wastage.
2. **Inappropriate repeated dispensing:** Pharmacists have their own share of medication wastage when they repeat the dispersal of medications. As a consequence of this inappropriate dispensing, patients may unintentionally use these medications along with other medications on hand, thereby overdosing themselves, leading to unnecessary side effects. Alternatively, patients may keep these repeated medications unused when they discover that the same medications are available at home. Marisa Domino et al. stated in a study conclusion, “The cost of drug therapy to North Carolina’s Medicaid program would probably increase if 34-day rather than 100-day supplies of medications were dispensed to patients” [35].
3. **Polypharmacy and Complex treatment regimens:** It is rational to prescribe medications as necessary to allow patients to appropriately address their medical conditions. Polypharmacy increases patients’ likelihood of adhering to their medications. Prescribing one or two of a medication per day encourages adherence [21]. Cognitively-impaired patients, such as senile patients, may present with self-harm symptoms when they over-adhere (by taking their prescribed medicines too frequently), and this harm could increase with polypharmacy prescriptions [21].
4. **Insufficient professional support for proper medicine use:** In some medical settings, pharmacists and physicians have high workloads with a short period of time allocated to each patient. This situation negatively affects their interactions with their patients and with each other. A reflection of this emerges when inadequate communication and support are given to patients, which can distract patients from medical care, and subsequently, the whole therapeutic process; this can lead to non-adherence to the medications given [33]. Patient insight should always be a focal point for healthcare practitioners; they should aim to understand the patients’ desires and then address them to increase patients’ adherence to medication. An example of that is when a side effect warning, such as “black box”, displayed on a medication pack deters patients from adherence, causing medication wastage [21]. A solution to such an obstacle is good communication between the healthcare provider and the patient, so that the patient knows that while it is imperative to mention the warning, it is unlikely to be applicable to their case. Patient reassurance has a remarkable impact on adherence [36].
5. **Treatment changes:** Trusting their prescribers, patients mostly follow their instructions even when they change their medications to more effective ones or to ones with fewer side effects. This change leads to the disuse of formerly prescribed medications [21]. The unused medications become a source of waste [4]. In many studies, changing therapies due to various reasons form a substantial waste of medications [37]. Braund reported “in percentage” that the reasons leading to medication disuse are medication expiry (26%), treatment change (24%), and condition reconciled (15%) [38]. West also ascribed medication wastage as being mainly due to “medication changes”, “patient death”, “resolution of patient’s condition”, “expired medication”, and overloading due to fear of medication inaccessibility [37].
6. **Long prescription duration:** In 1996, Hawksworth et al. determined that a drop in the cost of medications to a third of their total value would have occurred if these medications were dispensed for a period of 28 days [39]. The reason for this is that a patient who receives more than a month of medication may waste medication by changing therapy. Adherence rates are normally higher among patients with acute conditions, in contrast to those with chronic conditions [40]. Research papers uncovered that half of the medications endorsed for chronic sickness remained unused by patients [40].
7. **Over-sized Medication Package:** Patients consume a portion of the medicine supplied in oversized medication packages, and this is leads to waste [41,42]. Death of the patient, changes in prescription, excessive pack sizes, and repeat filling of prescriptions without assessing the amount at hand were all identified as major reasons why medicines may no longer be wanted and expire unused. In addition, patients may not proceed with medications after a treatment change by the prescribers or if they perceive their condition to have improved [40].
8. **Prescriber Financial Misalignment:** In a systematic review of the evidence, Wazana reported that promotions and deals affect doctors’ behaviors by pressuring them to increase pharmaceutical sales for financial interest [43].

#### 3.1.3. Regulation Causes

Absence or dis-implementation of available policies on the appropriate attitudes and behaviors of healthcare providers and patients can lead to malpractice in the form of irrational prescribing, irrational dispensing, and irrational use of medicines, which can broaden the level of medication wastage [44].

### 3.2. How Unwanted Medications are Disposed:

#### 3.2.1. Proper Disposal:

Unwanted medications are properly disposed of when they are returned to a specified location determined by the health authority. Optimistically, medication disposal systems are present in some countries, and there is a possibility of mimicking such systems in other regions of the world. Though there are no formal rules governing the return of unwanted medications, the public in New Zealand are encouraged to return their unwanted medications to the community pharmacies dispersed around the country [45]. However, the government of the United States, which is represented by the FDA and EPA (Environmental Protection Agency), has formulated a framework to deal with medication disposal [46]. Australian community pharmacies can receive unwanted medications for free through a program called (NatRUM), i.e., Return and Disposal of Unwanted Medicines, which was established in 1998 [47].

#### 3.2.2. Improper Disposal:

Unwanted medications may be improperly disposed of in the trash, sink, toilet, or given to a friend or a relative. In Kuwait, the public and pharmacists dispose of their unwanted medications in sinks or in the garbage [46]. The lack of an official program to control such wastage might play a role in this behavior, which was documented by 97% of study respondents. This raised the alarm for the need for quick actions [46]. Prescott et al. described the disposal of unwanted medications in commonly known places, such as the trash, sink, and toilet, as unsuitable, and stated that a return-back program should be introduced [46]. Pharmacists reported that they are not ready for the return of unwanted medications, and they are concerned about what to do with these items in the absence of relevant laws [46].

Table 1 below illustrates the details of some studies concerning medication wastage and disposal.

## 4. Discussion

In this narrative review, we attempted to qualitatively summarize articles and studies on unused medications, medication wastage, and medication disposal at home, and the reasons behind this disuse. This summary illustrates the situation of unused medications and may assist in developing new solutions to minimize medication wastage. According to the studies reviewed, the public, healthcare providers, media, and government are responsible for medication wastage. There are several factors that lead to general public waste of medication: multifactorial non-adherence, patient death, medication spoilage, medication loss, and stockpiling due to fear of medication shortage. On the other hand, healthcare providers are blamed for participating in the medication wastage problem through the repeated prescription and dispensing of medications, polypharmacy or complex treatment regimens, providing insufficient professional support to patients, providing long-duration prescriptions and over-sized medications, and changing therapies. Some people have the tendency to stockpile valuables, such as medications. If not used, these stockpiled pharmaceutical commodities will probably end up in the trash [4]. Governments contribute to medication wastage and improper disposal by not setting or not implementing policies and regulations to tackle these issues. The low awareness level of the public and the lack of systems in place to tackle this problem lead to the irrational disposal of medicines. As observed in most of the studies done in various nations, the public throw unwanted medications in the trash, sinks, and toilets, and rarely take them back to predetermined collection points, such as pharmacies [54]. However, as shown above, medication collection programs for safe disposal of unwanted medications are available in countries such as Australia and the United States. These programs could be adopted in other regions.

The use of medicines has dramatically increased and individuals seek remedies for even light symptoms that were formerly accepted. Efforts to scale back medication wastage should be sought, especially those linked to the main wastage source—the consumers [4]. People not only use medicines for therapeutic purposes, they also use them for social comfort and psychological reasons. Some of the demand for drugs probably arises because of the frail doctor–patient relationship or as a reflection of the naivety of the medical profession in curing illnesses and using science to determine the underlying causes of diseases. Medication wastage is a result of this rise in medication use. Patients should be encouraged to adhere to their medications primarily to optimize their health. By taking their medications as per the prescriber’s instructions, patients would play a great role in minimizing medication wastage. Patients and their carers should be advised to hand in unused and expired medications to designated collection points. Hospitals and clinics are perfect points for collecting large amounts of unwanted medication when death takes place by requesting the relatives of the dead turn in their unwanted at-home medications. The reduction of prescription supplies to a one-month supply may limit the wastage. To reduce medication costs without compromising a patient’s health, the use of generic alternatives should be encouraged among healthcare professionals after qualitatively evaluating these alternatives and their cost-effectiveness [50]. Lots of studies suggest that effective patient counseling and good pharmacist–patient relationships can have a great impact on the reduction of unwanted medication requests by patients [48]. In July 2018, Dawn Connelly said “Medicines reuse schemes successfully operate in the United States and Greece, reducing medicines waste and environmental pollution, as well as saving money and providing drugs to thousands of people who could not otherwise afford them” [55].

This review also found that awareness campaigns are needed to bring the economic and environmental consequences of medication wastage to the attention of the public. Above all, the therapeutic goals are not achieved if patients do not adhere to their medications, and therefore, these highly significant goals need to be addressed.

It has been noticed that some doctors may overprescribe medications due to pressure from their patients or sensitivity to the behaviors of others in their social networks. A phenomenon called “small-area variation” in healthcare practice refers to the fact that doctors in similar communities make treatment decisions simply based on the habits and practices of their peers. Pharmaceutical companies also influence what doctors prescribe. To curb the over-prescription of antibiotics, social benchmarking approaches work relatively well. Informing high prescribers that they are outliers compared to their peers results in a drop in their prescription rates. Prescriptions rates can also be lowered by the Nudge technique, where small doses are given at the beginning of treatment, and then the dose is tapered until the pain subsides. There are many mutual benefits for both patients and hospitals when the patients attending a hospital are invited to bring in their own medicines (POD i.e. Patient’s Own Drugs) from home. The utilization of POD and OSD (i.e. One-Stop Dispensing) may result in an efficient discharge and overall economic advantages. James et al. reported, “POD can reduce drug wastage from the destruction of patient’s medicines and eliminating complaints from patients and their general practitioners that medicines previously paid for are discarded” [56]. 

Pharmacists, particularly those armed with good communication and counseling skills, can directly and positively affect patient adherence to medications, therefore lessening medication wastage. Pharmacists are in a perfect position to convey valuable professional messages to consumers. They can advise patients on various medication issues, such as rational medication usage, in addition to medication wastage and proper disposal [46]. As technology has progressed to a great level, automated models like smart medicine cabinets should be studied and advanced to ease patient utilization and adherence to medications by reminding them to store medications under appropriate conditions and to alert patients about near-to-expiry medications.

Cooperation between all stakeholders, i.e., government institutes, organizations, the public, and healthcare professionals can, of course, help to minimize medication wastage through the implementation of practical policy and through increasing public and healthcare providers’ awareness.

## 5. Implications

The conclusions that all stakeholders (i.e., the public, healthcare providers, and the regulatory bodies) should work towards minimizing medication wastage can be summarized as follows:

- Awareness campaigns, education, and policies are cornerstones for all those contributing to medication wastage including the public, healthcare providers, and the government.
- Empowerment of patients to be accountable for adherence, medication wastage, and proper disposal of medication.
- Encouraging the public and healthcare providers by allocating incentives for those helping to reduce medication wastage and simultaneously penalizing those wasting medication if no persuading reason is declared.

Future research should explore this important topic of medication wastage and how to tackle it. We hope that this review contributes to the existing data and literature related to the topic of medication wastage.

## 6. Limitations

There is no doubt that some clear limitations are present in this narrative review. Firstly, the review only included English language articles, and thus, we might have omitted some related studies in other languages. Another limitation is that the review focused mainly on full-text papers retrieved from databases subscribed by our institution’s library.

## Figures and Tables

**Table 1:** Different studies on medication wastage and disposal.

Study Title Author/Country/Year	Objectives	Methodology	Findings
**1. Reasons Why Medicines Are Returned to Swedish Pharmacies Unused** [4]	To identify the reasons why medicines are returned to Swedish pharmacies unused and their relative importance	A semi-structured interview with pharmacy customers returning unused medicines to the pharmacy	Four reasons to return unused medicines to pharmacies: - The medicine was too old; - Patient death; - No need for medicine anymore; - Therapy changed. These reasons made up 75% of all reported reasons.
**2. Drug Consumers’ Behaviors toward the Disposal of Unused and Expired Medicines in Qassim Province, Saudi Arabia** [14]	To evaluate consumers’ knowledge about the disposal of unused medications and to determine the reasons for possessing unused medications and the types of unused medications.	Observational cross-sectional survey conducted in Saudi Arabia, between March 1 and April 1, 2017, using a pretested questionnaire.	Medications were mainly unused due to expiry. A total of 85.8% of respondents did not know the proper disposal method for unused and expired medicine; 57.72% keep medications at home until they expire; 28.52% flush them down the toilet; 5.03% give them to a friend. Two-thirds of respondents throw expired medications away.
**3. Identification of the reasons for medication returns** [1]	To identify and quantify the reasons for unused medications returned to pharmacies	Over a 5-week period, medications returned to two collection point pharmacies were analyzed for medication types and quantities. Those returning the medications were asked to complete a questionnaire to indicate why the medications were not used.	The main reasons indicated were “changed to other therapy” (37%) and “passed expiry date” (28%). There was one box of returns from an individual with a total calculated cost of over $14,500. The most commonly returned item was simvastatin, although half of the top 10 were “prn” or “as required” medications
**4. Practice, awareness, and opinion of pharmacists toward disposal of unwanted medications in Kuwait** [46]	To determine the practice of pharmacists, working in government healthcare sectors, with regard to disposal of returned unwanted medications by the public. To assess pharmacists’ awareness toward the impact of improper disposal on the environment and to investigate whether pharmacists agree to have their pharmacies as collection points for future take-back programs.	A random sample of 144 pharmacists from the six main governmental hospitals and 12 specialized polyclinics in Kuwait completed a self-administered questionnaire about their practice of disposal, awareness, and opinion on using pharmacies as collection points for proper disposal of Unwanted Medications. Data were analyzed using descriptive statistics.	A total of 144 pharmacists completed the survey. Throwing UMs in the trash was the main method of disposal by majority of the respondents (73%). Only 23 pharmacists disposed unwanted medications (UMs) according to the guidelines of Ministry of Health, Kuwait (MOH). However, about 82% are aware that improper disposal causes damage to the environment and 97% agree that it is their responsibility to protect the environment. About 86–88% of the pharmacists agree to have government hospital pharmacies and polyclinics as collection points for future take-back programs.
**5. An exploratory study on medications in Qatar homes** [17]	To characterize medications stored in Qatar homes and to explore their methods of storage and disposal, and to identify the public’s source of information related to medicines.	In this cross-sectional exploratory study, a list of telephone numbers was generated from Qatar’s telephone directory using a systematic sampling method. Individuals were interviewed using a multipart pretested survey.	Data was collected from 49 homes. Most respondents did not have a designated box for storing medications—48% kept drugs in their bedroom, and others kept them in the fridge or kitchen. The most commonly stored medicines were analgesics, antihistamines, nutritional supplements, and respiratory medications. Most people disposed of unwanted medicines by throwing them in the trash. In 15% of cases, the dosage was taken against instructions. Sharing of prescription medicines was not uncommon. The majority sought drug information from their doctor.
**6. An analysis of returned medicines in primary care** [16]	This pilot study was designed to investigate the types and amounts of medicines returned to both general practices (GPs) and associated local community pharmacies determining the reasons why these medicines have been returned	The study was conducted in eight community pharmacies and five GP surgeries within East Birmingham over a 4-week period.	A total of 114 returns were made during the study: 24 (21.1) to GP surgeries and 90 (78.9) to community pharmacies. The total returns comprised 340 items, of which 42 (12.4) were returned to GPs and 298 (87.6) to pharmacies, with the mean number of items per return being 1.8 and 3.3, respectively. Half of the returns in the study were attributed to the doctor changing or stopping the medicine; 23.7 of returns were recorded as excess supplies or clear out often associated with patients’ death and 3.5 of returns were related to adverse drug reactions. Cardiovascular drugs were most commonly returned, amounting for 28.5 of the total drugs returned during the study.
**7. Reducing Medicine Waste in the Community** [48]	- To make a measurable change in prescribed medicines with a reduction in medicine wastage. - To achieve improved standards of pharmaceutical care.	Information on patient needs and behavior came from pharmacy monitoring forms and interviews. The study compared outer and inner city populations. Participants were general practitioners, pharmacists, and 350 repeat prescription patients. Prescriptions were issued for two three-month periods.	Of the items prescribed, 23.8% were not dispensed, at a value of £13.1K, and 58% of the medications that were expected to be regularly supplied were collected.
**8. An Analysis of Unused and Expired Medications in Mexican Households** [23]	Expired medications were characterized according to the types of therapeutic groups, pharmaceutical dosage forms, expiration dates, and whether they were prescribed or over the counter drugs, and whether they came from Mexican health system or were purchased by the patients themselves.	The study was conducted in the metropolitan area of Monterrey during a 12-month period from March 2012 to February 2013. Unused and expired drugs were collected according to a program involving the collection and disposal of expired medications. Pharmacists and students from The Autonomous University of Nuevo León recorded data.	The number of medications classified was 22,140 items, corresponding to 30% of the total collected medications in that time; these were mostly NSAIDS (16.11%). Most were in solid form (73.39%) and were prescription drugs (91%) purchased at private pharmacies. The expiration date of medications ranged from 1995 to 2016, with 2011 being the outstanding year (36.66%).
**9. Returned Medicines: Waste or a Wasted Opportunity** [49]	To provide detailed data on the nature and scale of unused medicines in primary care, including GP surgeries as a disposal route, and the potential for re-use of these returned medicines	All medicines returned over two months to participating community pharmacies and GP surgeries in Eastern Birmingham primary care trust (PCT) were assessed for type, quantity, and value. A registered pharmacist assessed packs against set criteria to determine their suitability for possible re-use.	A total of 934 return events were made from 910 patients, comprising 3765 items worth £33,608. Cardiovascular drugs (1003, 27%) and central nervous system (CNS) drugs (884, 24%) were most prevalent. Returned packs had a median of 17 months remaining before expiry, and one-quarter of the packs (1248 out of 4291) were suitable for possible re-use. One-third of those suitable for re-use (476 out of 1248) contained drugs on the latest WHO Essential Drugs List.
**10. A Detailed Analysis of the Day to Day Unwanted Medicinal Products Returned to Community Pharmacies for Disposal** [39]	To provide realistic figures of day to day medicine wastage	Unwanted medicines are routinely returned, by the public, to community pharmacies for disposal. This is a routine service which community pharmacists offer for patients to dispose of unwanted medicines. There was no campaign to encourage the public to dispose of their unwanted medicines.	A total of 1091 items (dispensed containers containing medicinal products) valued at £7762 were returned during one month to 30 community pharmacies. This represents an annual wastage of £37.6 million. A total of 19.8% of the medicinal items were returned unopened. Death followed by end of prescription and having too much stock at home were the main reasons for return. The value of the medicines returned from patients was greater if the prescription was for more than 28 days.
**11. An Economic Assessment of the Extent of Medication Use and Wastage among Families in Saudi Arabia and Arabian Gulf Countries** [50]	To identify the extent of medication’s use and wastage among families in the Arabian Gulf countries, with an emphasis on Saudi Arabia.	A questionnaire was developed and administered to households in 5 regions in Saudi Arabia and other Gulf countries. A total of 1641 households participated in the study (1554 in Saudi Arabia; 87 in other countries).	The mean (SD) family size of household respondents from Saudi Arabia was 6.60 (3.20) members, with 0.32% reporting no medicines present in the household, 81.8% of households reporting 5 or more medicines, and 29.9% of respondents reporting having at least 10 medications at home. Overall, the mean (SD) number of medicines per household in Saudi Arabia was 8.0 (4.3). The most common therapeutic classes of medications kept at home in Saudi Arabia were respiratory medications (16.8%), CNS agents (16.4%), and antibiotics (14.3%). The mean (SD) number of drug products that were unused, deteriorated, or expired was 2.2 (2.7) and 2.7 (1.9) per household in Saudi Arabia and other Gulf countries, respectively. The mean medication wastage was 25.8% (Saudi Arabia) and 41.3% (other Gulf countries). When analyzed on the basis of total medication cost, medication wastage was 19.2% and 25.0% in Saudi Arabia and other Gulf countries, respectively. The mean out-of-pocket expenditure (based on the percentage of annual income) for medications was 0.72% for households in Saudi Arabia, compared with 0.48% in other Gulf countries.
**12. Analysis of medications returned to community pharmacies** [51]	To identify and quantify the types and amounts of medications returned to community pharmacies, and specifically, to quantify the percentage of medication returned from the original dispensing, its therapeutic category, and reasons for not being used.	Unsolicited medication returned for disposal to the 24 community pharmacies in the Taranaki region (approximately 37,000 households) of New Zealand over a 6-week period was analyzed. The results were entered into a database, recording medication, amount originally issued (if known), date of issue, Anatomical Therapeutic Chemical (ATC) classification, and reason for nonuse. Cross-tabulation of ATC category versus percentage returned and ATC category versus reason for returns was performed. Adjusted standardized residuals were investigated to determine specific cells that were in excess of the expected counts.	Complete information was available for 2704 items. The majority (51%) of returns contained 75–100% of the original dispensed amount of medication. For the respiratory category, 77% of the returns were in the 75–100% group, significantly more than for any other therapeutic group. Reasons for returns were recorded as bereavement (22%), surplus to requirements (17%), expired (8%), medication change (11%), dose change (3%), and unknown (39%). The cardiovascular group and respiratory groups had a higher rate of returned drugs due to medication changes and surplus to requirements, respectively.
**13.Pharmacists’ Activities to Reduce Medication Waste: An International Survey** [20]	To identify activities that pharmacists undertake to reduce medication waste, and to assess the extent to which these activities are implemented, their importance for waste-reduction, and feasibility for broad implementation	A two-phase survey was conducted among community and hospital pharmacists working in different developed countries. Phase one used an open-ended questionnaire to identify activities undertaken by pharmacists. Answers were thematically analyzed to construct a list of medication waste-reducing activities. In phase two, a questionnaire was disseminated among pharmacists from different countries, to assess if these activities are implemented (yes/no), their importance, and feasibility (1 to 5 ranking scale).	In phase one, 53 pharmacists participated and 14 activities were identified. These were categorized into the pharmaceutical supply chain: prescribing, dispensing (pharmacy or patient-related), and leftover stages. In phase two, 89 pharmacists participated. Most activities were implemented by a minority of pharmacists. Reducing medication amounts in stock was most frequently implemented (dispensing stage pharmacy-related; 86%), followed by collecting unused medications (leftover stage; 77%) and performing a medication review (dispensing stage; 68%). Waste-reducing activities in the dispensing stage activities were both considered most important and feasible (ranked 4). Overall, most activities scored higher on importance than on feasibility.
**14. Patient-Related Risks for Non-adherence to Antiretroviral Therapy among HIV-Infected Youth in the United States: A Study of Prevalence and Interactions** [24]	To determine the prevalence of personal barriers to adherence and to identify associations between these barriers in HIV-infected subjects	A cross-sectional, observational study. We studied the following personal barriers to adherence: mental health barriers, high or low self-efficacy and outcome expectancy, and the presence of specific structural barriers	There were 396 subjects infected after age 9 recruited from sites from the Adolescent Trials Network for HIV/AIDS Interventions or the Pediatric AIDS Clinical Trials Group. Of the 396 subjects, 148 (37.4%) self-identified as non-adherent. No significant differences were found between adherent and non-adherent subjects for the presence of mental health disorders. Adherence was significantly associated with all but one structural barrier. Both self-efficacy and outcome expectancy were higher among adherent versus non-adherent subjects (p < 0.0001). Grouping subjects according to low self-efficacy and outcome expectancy for adherence, adherence differed according to the presence or absence of mental health disorders and structural barriers (p < 0.0001). Our data suggest that adolescents have significant rates of non-adherence and face multiple personal barriers.
**15. Long-term adherence with cardiovascular drug regimens** [26]	To characterize adherence to evidence-based cardiovascular medications prescribed at hospital discharge at 1 year.	We studied 1326 patients with coronary artery disease undergoing cardiac catheterization between 1998 and 2001. We examined adherence to angiotensin-converting enzyme (ACE) inhibitors, aspirin, beta-blockers (BBs), and statins by comparing baseline prescription at hospital discharge to self-reported medical regimen at 12 months. Patients who reported use of each cardiac medication at 1 year were considered adherent. Clinical and demographic predictors of nonadherence are described.	The population had a mean age of 65.7 +/- 10.5 years, and 36% were women. At discharge, aspirin was prescribed in 95%, BBs in 86%, ACE inhibitors in 65%, and statins in 55%. The proportion of patients who discontinued medications was lowest for aspirin (18%) and BBs (22%), and highest for ACE inhibitors and angiotensin receptor blockers (28%) and statins (28%). Only 54% were adherent to all of their initial medications. Patients who discontinued medications were more likely to be older, women, unmarried, and less educated. Multivariable predictors of better adherence were higher mental health, education level, marital status, and no antidepressant use. A higher number of prescribed medications were associated with lower adherence to the recommended regimen. Insurance coverage and physical function did not correlate with adherence.
**16. Study by Alberta pharmacists indicates drug wastage a “mammoth” problem** [28]	To know the reasons why people do not take their medicines.	For several years Albertans have been urged to return unused medicines to pharmacists. Collected medicines were studied in 58 pharmacies for 8 weeks.	Reasons for not taking medicines: 26.6% patient death, less than 25% due expired medicines, then due to physician-directed change and feeling better (11%), allergic reactions 8%, did not want to take the drug (7%). More than 10% of the drugs returned were more than 5 years old, 20% were over-the-counter products; 72.4% were prescription drugs. People making returns brought back an average of 60% of the drugs in the original prescription. More than 6% of returned drugs came from physicians’ offices as samples.
**17. Restricting patients’ medication supply to one month: saving or wasting money?** [35]	State Medicaid program’s pharmacy expenditures associated with dispensing one- and three-month supplies of drugs were examined	We simulated the effect of a policy change from a maximum of a 100-day supply of prescription medication to one where only a 34-day supply was allowed. All North Carolina prescription claims from Medicaid enrollees who filled a prescription for at least one of six medication categories during fiscal years 1999 and 2000 were included. The six categories were angiotensin-converting-enzyme inhibitors, anti-ulcers, antipsychotics, non-steroidal anti-inflammatory drugs, selective serotonin-reuptake inhibitors, and sulfonylureas. The dollar value of the medication wasted, the amount of medication wastage diverted after a change to a shorter prescription length, and the total costs incurred by the increases in prescription refills were calculated.	For each therapeutic category, 255,000–783,000 prescription drug claims were analyzed. No valid drug claims were excluded for any reason. Although 5–14% of total drug wastage, attributed to switches of drug therapy, could be saved by dispensing a 34-day supply, this saving could not make up for a larger increase in dispensing costs, as consumers would fill prescriptions more often. In addition, reducing the amount of drug dispensed each time may be costly to consumers through increased transportation and other expenses.
**18. Investigating unused medications in New Zealand** [38]	To determine the reasons for returning medications unused and the types of unused medications returned based on therapeutic class	A “Disposal of Unwanted Medication Properly (DUMP)” campaign was conducted for a 4-week period in November 2007 in the Hutt Valley DHB region. A collection bag was delivered to every household for the collection and disposal of any unused medications. Participants were instructed to return the bags to a community pharmacy. Those returning medications were also asked to complete a questionnaire to determine why the medications were not used. A sample of the returned medications was identified, quantified and every completed questionnaire was analyzed.	Over the 4-week period, 1605 bags were returned for disposal. A total of 329 bags (20%) containing a total of 1253 items were fully analyzed. Only 653 questionnaires were completed (41%) all of which were analyzed. The most commonly reported reason for not using the medication was that it had passed the expiry date (26%), the second was treatment change (24%), followed by condition resolved (15%). “Alimentary tract and metabolism” and “respiratory systems and allergies” accounted for 21 and 20% of cost, respectively.
**19. Medicines discarded in household garbage: Analysis of a pharmaceutical waste sample in Vienna** [42]	To analyze a sample of pharmaceutical waste drawn from household garbage in Vienna, with the aim to learn whether and which medicines end up unused in normal household waste.	We obtained a pharmaceutical waste sample from the Vienna Municipal Waste Department. This was drawn by their staff in a representative search in October and November 2009. We did a manual investigation of the sample which contained packs and loose blisters, excluded medical devices, and traced loose blisters back to medicines packs. We reported information on the prescription status, origin, therapeutic group, dose form, contents, and expiry date. We performed descriptive statistics for the total data set and for sub-groups (e.g., items still containing some of original content).	In total, 152 packs were identified, of which the majority was prescription-only medicines (74%). Cardiovascular medicines accounted for the highest share (24%); 87% of the packs were in oral form; 95% of the packs had not expired; 14.5% of the total data set contained contents but the range of content left in the packs varied. Results on the packs with contents differed from the total—the shares of Over-the Counter medicines (36%), of medicines of the respiratory system (18%) and of the musculo-skeletal system (18%), for dermal use (23%), and of expired medicines (19%) were higher compared to the full data set.
**20. Disposal practices for unused medications around the world** [45]	To survey the current peer-reviewed literature on attitudes and practices to medicine disposal methods as reported by patients and the various medication disposal and destruction systems around the world.	A literature search was carried out using the keywords “medicines disposal”, “unused medicines”, “medicines wastage”, and “medication disposal” in the PubMed TM, ISI Web of Knowledge TM, Google Scholar TM, Medline TM, Scopus TM, and International Pharmaceuticals Abstracts TM up until the end of May 2010. Twelve peer-reviewed articles with specified sample sizes were selected	The most popular methods for medication disposal were in the garbage, toilet, or sink. Liquid medications were more likely to be rinsed down the sink, as opposed to solid tablets and capsules, which were more likely deposited in the rubbish bin. Much confusion exists about the “proper” way of medication disposal as many countries do not have standard medication disposal protocols. Furthermore, some pharmacies around the world refused to accept unused medications or discouraged the practice.
**21. Patient Practices and Beliefs Concerning Disposal of Medications** [52]	To survey patient practices and beliefs concerning disposal of medications.	A total of 301 patients at an outpatient pharmacy completed a survey about medication disposal practices and beliefs.	More than half of the patients surveyed reported storing unused and expired medications in their homes, and more than half had flushed them down a toilet. Only 22.9% reported returning medication to a pharmacy for disposal. Less than 20% had ever been given advice about medication disposal by a healthcare provider. Previous counseling was highly associated with returning medications to a pharmacy (45.8% vs. 17.1%, P < 0.001) and was the variable most associated with returning medications to a provider (28.8% vs. 10.0%, P < 0.001). Previously counseled respondents were significantly more likely to believe that returning medications to a pharmacy (91.5% vs. 60.3%, P < 0.001) or a medical provider (74.6% vs. 47.3%, P < 0.001) was acceptable.
**22. Taking Stock of Medication Wastage: Unused Medications in U.S. Households** [53]	To estimate the extent, type, and cost of unused medications and the reasons for their nonuse among U.S. households.	A cross-sectional, observational two-phased study was conducted using a convenience sample in Southern California. A web-based survey (Phase I, *n* = 238) at one health sciences institution and paper-based survey (Phase II, *n* = 68) at planned drug take-back events at three community pharmacies were conducted. The extent, type, and cost of unused medications and the reasons for their nonuse were collected.	Approximately 2 out of 3 prescription medications were reported unused; disease or condition improved (42.4%), forgetfulness (5.8%), and side effects (6.5%) were reasons cited for their nonuse. “Throwing medications in the trash” was found being the common method of disposal (63%). In phase I, pain medications (23.3%) and antibiotics (18%) were most commonly reported as unused, whereas in Phase II, 17% of medications for chronic conditions (hypertension, diabetes, cholesterol, heart disease) and 8.3% for mental health problems were commonly reported as unused. Phase II participants indicated pharmacy as a preferred location for drug disposal. The total estimated cost for unused medications was approximately $59,264.20 (average retail Rx price) to $152,014.89 (AWP) from both phases, borne largely by private health insurance. When extrapolated to a national level, it was approximately $2.4B for elderly taking five prescription medications to $5.4B for the 52% of US adults who take one prescription medication daily.
**23. Knowledge and Barriers to Safe Disposal of Pharmaceutical Products Entering the Environment** [54]	To examine the potential correlations between people’s actual disposal practices and their knowledge of the impact of disposal practices on the environment and human health, and availability of disposal options.	A quantitative cross-sectional study. Respondents to an online survey were 485 residents of the northeast United States, polled from the general population. Descriptive statistics and logistic regression were used to model responses from the dependent variable actual disposal practice (ADP) across the independent variables, and analysis of variance explored whether ADP differed across demographic variables. The conceptual framework selected for this study comprised 2 models: the health belief model and the theory of planned behavior	Statistically significant associations emerged among individuals’ knowledge of environment and human-health impact, recommended disposal practices, disposal options, and that person’s likelihood to practice recommended disposal. Demographic variables did not impact disposal behavior.
**24. Reviewing repeat prescribing—General practitioners and community pharmacists working together** [34]	To determine the potential role that community pharmacists could fulfill in reviewing and rationalizing medication received on repeat prescriptions.	Volunteer pharmacists and general practitioners in two health authorities in England formed 47 GP-pharmacist partnerships. Each GP identified up to 50 patients receiving six or more “repeat” medicines. Pharmacists reviewed GP notes and record systems to identify potential problems. Discussions addressing the identified problems and possible solutions were held between the GP-pharmacist partners. Three months later the pharmacists revisited the surgeries to review GP notes to determine whether changes had occurred. On completion of the project, focus groups were held with participating GPs and pharmacists to ascertain their views on the project	In total, repeat prescriptions for 1564 patients were investigated. which resulted in 13,194 medicines being reviewed. The pharmacists identified 9762 potential “problems”, of which the most common were: drugs no longer required, inappropriate quantity ordered, and unsatisfactory directions. The GPs agreed with 58 per cent of identified problems and acted upon 56 per cent of these (32 per cent of the original total). In general, the involvement of pharmacists was beneficial in terms of rationalizing prescribing and reducing possible drug interactions and adverse drug reactions resulting from multiple supplies of potentially inappropriate and unnecessary medication. Furthermore, pharmacists were able to rationalize patients’ supplies to help improve the actual management of medication in terms of ordering and collecting supplies and coping with complex daily regimens.
**25. A systematic review of the literature on “medication wastage”: An exploration of causative factors and effect of interventions** [3]	To systematically review the published literature, the possible causative factors associated with medication wastage and the effectiveness of any interventions to reduce wastage.	A systematic review of studies published in English was identified from the following databases: Cumulative Index to Nursing and Allied Health Literature, Embase, Medline, PubMed, Science Citation Index, and The Cochrane Library. Data extraction and critical appraisal was undertaken independently by two researchers.	Title, abstract and full paper screening reduced the 14,157 studies to 42. A general definition of medication wastage was reported in one paper only. “Medication changed”, “patient death”, “resolution of patient’s condition”, and “expired medications” were most commonly cited reasons for wastage. Only two studies were identified reporting wastage as a research outcome measure following intervention.
